# Differential effects of plant protein ratio on renal function and mortality across CKD stages

**DOI:** 10.3389/fnut.2025.1596836

**Published:** 2025-07-28

**Authors:** Guoyi Wang, Qiong Yi, Xueqin Zhang, Min Zhou, Jinwen Zhao, Haiyuan Lu, Ju Li, Deqian Meng, Yong Xu, Kai Wang

**Affiliations:** ^1^Department of Nephrology, The Affiliated Huaian No.1 People’s Hospital of Nanjing Medical University, Huaian, China; ^2^Department of Rehabilitation, The Affiliated Huaian No.1 People’s Hospital of Nanjing Medical University, Huaian, China; ^3^Department of Rheumatology and Immunology, The Huaian Clinical College of Xuzhou Medical University, Huaian, China; ^4^Department of Rheumatology and Immunology, The Affiliated Huaian No.1 People’s Hospital of Nanjing Medical University, Huaian, China

**Keywords:** chronic kidney disease, plant protein, mortality, estimated glomerular filtration rate, dietary protein

## Abstract

**Background:**

While plant protein has been suggested to offer renoprotective benefits, the optimal proportion of dietary plant protein and its relationship with outcomes across different stages of chronic kidney disease (CKD) remains unclear.

**Methods:**

Using data from the National Health and Nutrition Examination Survey (NHANES), we examined the association between plant protein ratio and estimated glomerular filtration rate (eGFR) across CKD stages. Plant protein ratio was categorized as low (< 33%), medium (33%–66%), and high (≥ 66%). Multiple imputation was performed for missing data. Weighted linear regression models were used to analyze plant protein ratio-eGFR associations, while Cox proportional hazards models assessed mortality risk. Dose-response relationships were evaluated using restricted cubic splines.

**Results:**

Among 16,163 participants, distinct patterns emerged across CKD stages. In Non-CKD, high plant protein ratio was associated with significantly higher eGFR compared to low plant protein ratio (β = 0.790, *P* = 0.039). In CKD G4, medium plant protein ratio showed significantly higher eGFR (β = 1.791, *P* = 0.025) compared to low plant protein ratio. For mortality risk, CKD G3 patients with medium plant protein ratio demonstrated significantly lower risk (HR = 0.67, 95% CI: 0.44–1.00, *P* = 0.047) compared to low plant protein ratio. Dose-response analyses revealed stage-specific patterns: U-shaped relationships in early CKD, transitioning to inverted U-shaped and J-shaped patterns in advanced stages.

**Conclusion:**

The association between plant protein ratio and outcomes varies across CKD stages, suggesting the need for stage-specific dietary recommendations. While moderate plant protein intake might be beneficial in early CKD, our findings in advanced stages were largely non-significant and require confirmation in larger studies before clinical recommendations can be made. These findings support a more nuanced approach to dietary protein source management in CKD, though further prospective studies are needed to confirm these associations.

## Introduction

Chronic kidney disease affects approximately 10% of the global population and is a leading cause of morbidity and mortality worldwide, with projections indicating a 30% increase in prevalence by 2040 (1). Dietary management, particularly protein intake optimization, remains a cornerstone of CKD care to delay disease progression and mitigate complications. Current guidelines from the Kidney Disease: Improving Global Outcomes (KDIGO) recommend limiting protein intake to 0.6–0.8 g/kg/day for advanced CKD patients (2). However, emerging evidence suggests that protein source–specifically plant versus animal origin–may exert differential effects on renal outcomes (3–5). Plant-based proteins are hypothesized to reduce uremic toxin production, improve acid-base balance, and lower phosphorus load, potentially offering renoprotective advantages (6–8).

The potential benefits of plant-based diets in nephrology have been demonstrated by several recent studies. A 2024 meta-analysis of 18 randomized controlled trials revealed that soy protein intake showed protective effects against hyperlipidemia and proteinuria in CKD patients (9). Furthermore, a prospective cohort study found that higher plant protein intake was associated with a 71% lower odds of incident CKD (OR = 0.29, 95% CI: 0.15–0.55), independent of total protein consumption (10). The ARIC study (Atherosclerosis Risk in Communities) provided additional evidence by demonstrating differential associations between protein sources and CKD risk: while red and processed meat consumption increased CKD risk, nuts, low-fat dairy products, and legumes showed protective effects (11).

While previous studies have provided valuable insights into protein intake and CKD outcomes, several critical knowledge gaps remain that limit clinical translation. Existing research has primarily focused on absolute protein intake rather than the proportion of plant-to-animal protein sources, which may be more clinically relevant for dietary counseling. For instance, a multicohort analysis demonstrated protective effects for both plant and animal protein against CKD progression (per 0.2 g/kg/d increment: HR = 0.80 for plant protein vs. HR = 0.88 for animal protein) (12), and a comprehensive meta-analysis of 148,051 participants showed inverse associations with CKD incidence for total protein (RR = 0.82), plant protein (RR = 0.77), and animal protein (RR = 0.86) (13). However, these studies did not address the optimal balance between protein sources or examine stage-specific effects across the CKD spectrum.

Most importantly, no previous study has systematically examined how plant protein ratio affects both renal function and mortality outcomes across different CKD stages using dose-response analyses. This represents a significant gap in our understanding, as the metabolic demands, protein utilization efficiency, and cardiovascular risk profiles vary substantially across CKD stages. Furthermore, while absolute protein intake recommendations exist for CKD patients, evidence-based guidance on the optimal proportion of plant versus animal protein sources remains lacking, particularly for different disease stages. The National Health and Nutrition Examination Survey (NHANES) provides a unique opportunity to investigate these relationships in a nationally representative sample. This study aims to investigate the association between plant protein ratio and renal function across different CKD stages, while also examining dose-response relationships and mortality risk.

## Materials and methods

### Study population and data collection

This cross-sectional study utilized data from the NHANES database, employing a multistage stratified sampling design to investigate the association between plant-based protein proportion and renal function [measured by estimated glomerular filtration rate (eGFR)] in CKD patients. Implemented Multiple Imputation by Chained Equations with predictive mean matching for key variables, generating 5 imputed datasets (percentage of missing data see Supplementary Figure 1).

### Dietary assessment

Dietary data were collected through 24-h dietary recalls administered by trained interviewers using the USDA’s Automated Multiple-Pass Method. The first recall was conducted in-person, and the second recall was completed via telephone 3–10 days later. Protein intake was categorized into plant and animal sources based on food codes. Protein sources were classified using dietary recall data, with plant protein ratio defined as the percentage of plant-based protein (legumes, nuts, seeds, and grains; for a comprehensive list of plant protein source classifications, see Supplementary Figure 2) relative to total protein intake, categorized into three groups: low (< 33%), medium (33%–66%), and high (≥ 66%). Total energy intake was calculated as kilocalories per day.

### Laboratory measurements

Blood samples were collected after an overnight fast of at least 8 h. All laboratory tests were performed at Collaborative Laboratory Services (Ottumwa, Iowa) using a Beckman Colter DxC800 analyzer. eGFR was calculated using the CKD-EPI equation: eGFR = 141 × min(Scr/κ, 1)α × max(Scr/κ, 1) −1.209 × 0.993Age × 1.018[if female] × 1.159[if black] where Scr is serum creatinine, κ is 0.7 for females and 0.9 for males, and α is −0.329 for females and −0.411 for males.

### Statistical analysis

Weighted linear regression models accounting for complex survey design were employed to analyze the association between plant protein ratio and eGFR in CKD patients. For this analysis, plant protein ratio was categorized into three groups: low (< 33%), medium (33%–66%), and high (≥ 66%), with the low group serving as the reference. Results are reported as β coefficients and 95% confidence intervals (CIs).

Cox proportional hazards models were used to assess the association between plant protein ratio and mortality risk in CKD patients. The same categorization of plant protein ratio was applied (low, medium, and high groups). The models incorporated survival time (follow-up duration) and death status as outcome variables, with results reported as hazard ratios (HRs) and 95% CIs.

The dose-response relationships between plant protein proportion and both eGFR and all-cause mortality across CKD stages were evaluated using restricted cubic splines (RCS) with three knots. Plant protein proportion was treated as a continuous variable (0%–100%) with extreme values Winsorized at the 1st and 99th percentiles. For each CKD stage, we accounted for the complex survey design of NHANES by incorporating stratification variables, clustering units, and sampling weights. Three separate generalized linear models were fitted across imputed datasets using the svyglm function from the survey package. Non-linear relationships were modeled using restricted cubic splines with knot locations determined by Harrell’s recommended percentiles (10%, 50%, 90%). Non-linearity was tested through likelihood ratio tests comparing models with linear terms against those containing spline components. The proportional hazards assumption was verified using Schoenfeld residuals. Adjusted predictions were generated across the plant protein spectrum (1%–100%) while holding covariates at referent values: male gender, white ethnicity, median age, median Body Mass Index (BMI), and median energy intake. 95% CIs were calculated using Rubin’s rules for multiple imputation pooling.

To account for disease heterogeneity, separate models were constructed for different CKD stages (Non-CKD, G2-G5). All models were adjusted for potential confounders including age (continuous), sex (categorical), race/ethnicity (categorical), BMI (continuous), total energy intake (continuous, kcal/day), and blood glucose (continuous, mg/dL). To account for NHANES’ complex survey design, appropriate survey weights, strata, and cluster variables were incorporated into all analyses. The weighted estimates are representative of the non-institutionalized US civilian population.

Statistical significance was set at *P* < 0.05. All analyses were performed using R version 4.1.0 (R Foundation for Statistical Computing, Vienna, Austria) with the following packages: mice for multiple imputation, survey for complex survey analysis, and ggplot2 for visualization.

## Results

### Baseline characteristics of study participants

**FIGURE 1:**
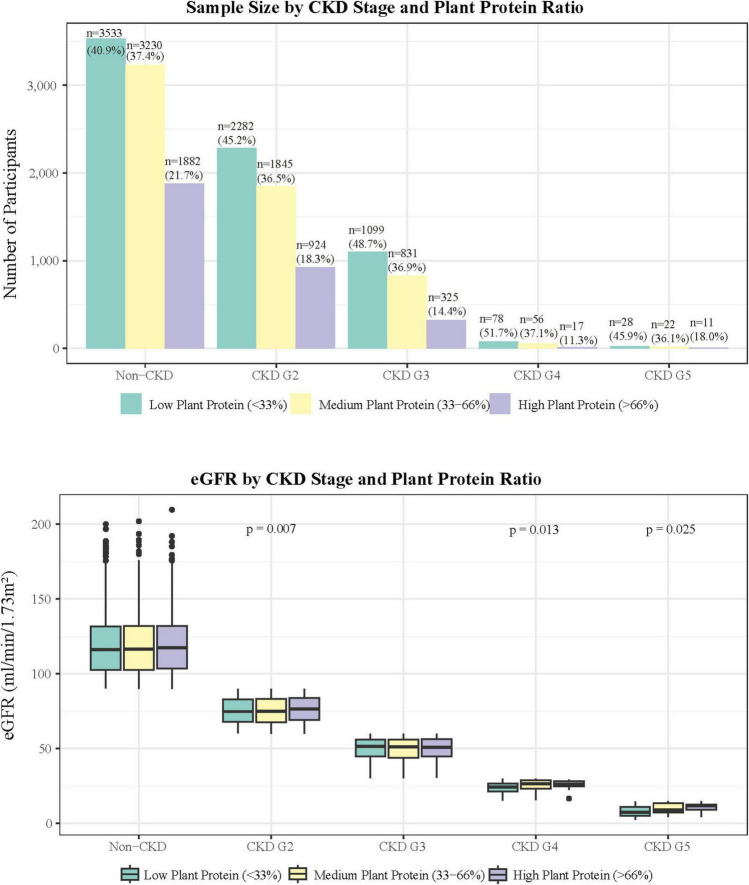
Plant protein intake patterns and renal function stratification: distribution of eGFR and population characteristics across chronic kidney disease stages.

The study population comprised 16,163 participants distributed across different CKD stages (Figure 1A). In the Non-CKD group (*n* = 8,645), 40.9% consumed low plant protein, 37.4% medium plant protein, and 21.7% high plant protein. Similar distribution patterns were observed in CKD G2 (*n* = 5,051) with 45.2%, 36.5%, and 18.3%, respectively. The proportion of participants gradually decreased in advanced CKD stages, with CKD G5 having the smallest sample size (*n* = 61).

The relationship between plant protein ratio and eGFR varied across CKD stages (Figure 1B). In the Non-CKD group, eGFR levels were comparable among different plant protein ratios (low: 118.3 ± 19.0, medium: 118.5 ± 18.9, high: 118.9 ± 18.9 ml/min/1.73 m^2^; *p* = 0.422). In CKD G2, participants with high plant protein ratio demonstrated significantly higher eGFR compared to other groups (76.1 ± 8.6 vs. 75.1 ± 8.6 vs. 75.2 ± 8.8 ml/min/1.73 m^2^; *p* = 0.007), with *post hoc* analysis confirming differences between high vs. low (*p* = 0.0067) and high vs. medium (*p* = 0.0216) groups.

No significant differences were observed in CKD G3 (low: 49.7 ± 7.6, medium: 49.2 ± 8.0, high: 49.6 ± 7.7 ml/min/1.73 m^2^; *p* = 0.574). However, in CKD G4, both medium and high plant protein groups showed significantly higher eGFR compared to the low plant protein group (25.6 ± 3.6 vs. 25.5 ± 3.1 vs. 23.9 ± 3.8 ml/min/1.73 m^2^; *p* = 0.013). Similarly, CKD G5 demonstrated a significant association (*p* = 0.025), with an ascending trend in eGFR across plant protein ratios (low: 7.7 ± 3.7, medium: 10.0 ± 3.6, high: 10.6 ± 3.4 ml/min/1.73 m^2^).

Age distribution showed an increasing trend with CKD progression, ranging from 42.5 ± 16.5 years in Non-CKD to 72.5 ± 10.1 years in CKD G4. BMI remained relatively stable across groups (range: 27.9–32.8), with the highest values observed in the high plant protein group of CKD G4 (32.8 ± 6.8) and lowest in the medium plant protein group of CKD G5 (27.9 ± 7.5).

The proportion of participants consuming high plant protein (≥ 66%) decreased with advancing CKD stages, from 21.7% in Non-CKD to 18.0% in CKD G5. This trend was consistent across all CKD stages, with the lowest proportion observed in CKD G4 (11.2%).

### Association between plant protein ratio and eGFR in CKD patients

After adjusting for potential confounders using weighted linear regression models that accounted for complex survey design, the association between plant protein ratio and eGFR showed stage-specific patterns. In the Non-CKD group (Supplementary Figure 3), Participants with a high plant protein ratio (≥ 66%) demonstrated a statistically significant increase in eGFR compared to the low ratio group (< 33%) (β = 0.79, *P* = 0.039). However, no significant difference was observed between the medium (33%–66%) and low ratio groups (β = 0.333, *P* = 0.359).

**FIGURE 2:**
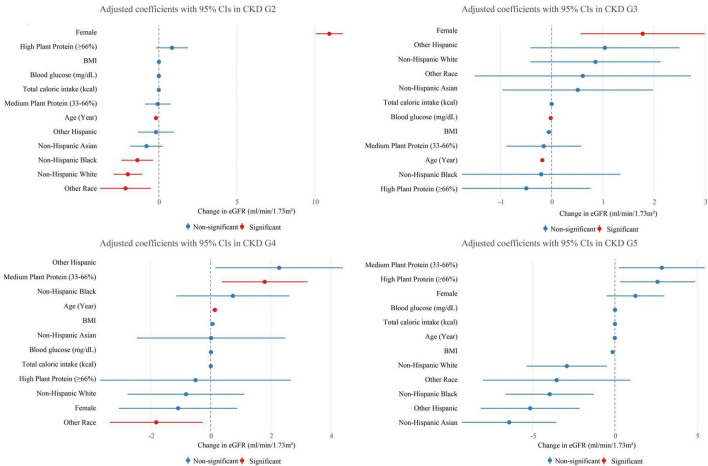
Association between plant protein ratio and eGFR in CKD (G2-G5) patients.

In CKD G2 (Figure 2A), no significant association was observed between high plant protein ratio and eGFR (β = 0.852, *P* = 0.113)., while the medium ratio showed no association with eGFR (β = −0.068, *P* = 0.872). Notably, female gender (β = 10.943, *P* < 0.001) and younger age (β = −0.18 per year, *P* < 0.001) were significantly associated with higher eGFR in this stage.

For CKD G3 (Figure 2B), no statistically significant associations were observed between plant protein ratio and eGFR (medium: β = −0.155, *P* = 0.681; high: β = −0.495, *P* = 0.445). Key predictors included female gender (β = 1.777, *P* = 0.007) and lower blood glucose levels (β = −0.018 per mg/dL, *P* < 0.001).

In CKD G4 (Figure 2C), the medium plant protein ratio was significantly associated with higher eGFR (β = 1.791, *P* = 0.025), whereas the high ratio group showed no significant effect (β = −0.508, *P* = 0.758). A paradoxical positive association between age and eGFR was observed (β = 0.135 per year, *P* = 0.005), potentially reflecting survival bias in advanced CKD.

For CKD G5 (Figure 2D), no statistically significant associations were observed between plant protein ratio and eGFR (medium: β = 2.843, *P* = 0.112; high: β = 2.583, *P* = 0.189). Racial/ethnic disparities were prominent, with Other Hispanic (β = −5.145), Asian (β = −6.420), and Black (β = −3.960) participants exhibiting lower eGFR compared to the reference group (White), though statistical significance was not reported.

### Association between plant protein ratio and mortality risk in CKD patients

In the analysis of all-cause mortality using Cox proportional hazards models, the association between plant protein ratio and mortality risk varied across CKD stages. In the Non-CKD group (Supplementary Figure 4), neither medium (HR = 1.29, 95% CI: 0.75–2.22, *p* = 0.349) nor high plant protein ratio (HR = 1.46, 95% CI: 0.83–2.54, *p* = 0.188) showed significant associations with mortality risk. However, age (HR = 1.05, 95% CI: 1.04–1.07, *p* < 0.001) and race/ethnicity were significant predictors of mortality in this group.

**FIGURE 3:**
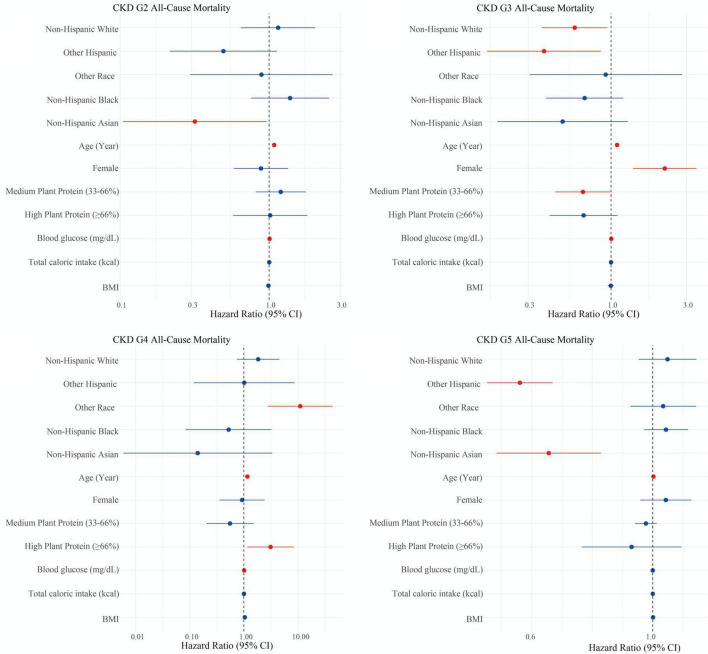
Association between plant protein ratio and mortality risk in CKD (G2-G5) patients. Results for CKD G5 should be interpreted with extreme caution due to small sample size (*n* = 61) and wide confidence intervals.

For CKD G2 patients (Figure 3A), plant protein ratio showed no significant association with mortality risk (medium: HR = 1.20, 95% CI: 0.81–1.76, *p* = 0.366; high: HR = 1.01, 95% CI: 0.57–1.80, *p* = 0.960). Asian race showed a protective effect against mortality (HR = 0.32, 95% CI: 0.10–0.96, *p* = 0.042), while age (HR = 1.08, 95% CI: 1.06–1.10, *p* < 0.001) and blood glucose levels (HR = 1.01, 95% CI: 1.00–1.01, *p* = 0.003) were associated with increased mortality risk.

In CKD G3 (Figure 3B), medium plant protein ratio was associated with a borderline significant reduction in mortality risk (HR = 0.67, 95% CI: 0.44–1.00, *p* = 0.047) compared to low plant protein ratio. No statistically significant association was observed for the high plant protein ratio group (HR = 0.67, 95% CI: 0.41–1.1, *p* = 0.113). Female gender was associated with increased mortality risk (HR = 2.18, 95% CI: 1.38–3.46, *p* = 0.001), while Other Hispanic (HR = 0.38, 95% CI: 0.16–0.86, *p* = 0.021) and White race (HR = 0.59, 95% CI: 0.37–0.95, *p* = 0.030) showed protective effects.

For CKD G4 (Figure 3C), high plant protein ratio was associated with significantly increased mortality risk (HR = 3.10, 95% CI: 1.15–8.31, *p* = 0.025), while medium plant protein ratio showed no significant association (HR = 0.55, 95% CI: 0.20–1.50, *p* = 0.246). Age (HR = 1.16, 95% CI: 1.05–1.27, *p* = 0.002) and Other race category (HR = 10.93, 95% CI: 2.77–43.16, *p* = 0.001) were associated with increased mortality risk.

In CKD G5 (Figure 3D), no statistically significant associations were observed between plant protein ratio and mortality risk (medium: HR = 0.46, 95% CI: 0.13–1.56, *p* = 0.21; high: HR = 0.09, 95% CI: 0.00–26.59, *p* = 0.406). Other Hispanic ethnicity showed a protective effect (HR = 0.57, 95% CI: 0.45–0.68, *p* = 0.003), while age remained a significant predictor of mortality (HR = 1.08, 95% CI: 1.00–1.17, *p* = 0.042).

### Dose-response relationship between plant protein ratio and eGFR in different CKD stages

**FIGURE 4:**
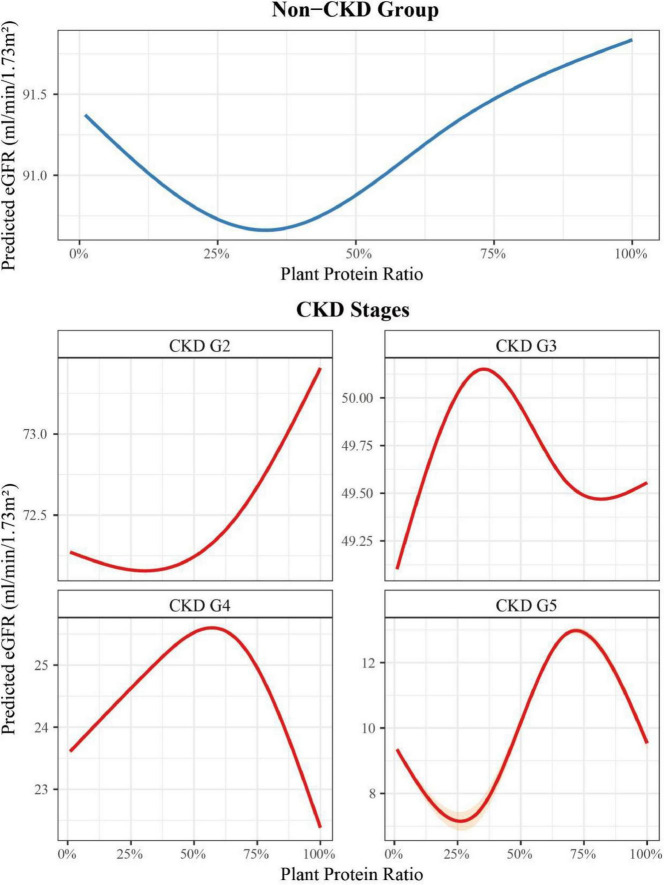
Dose-response relationship between plant protein ratio and eGFR across CKD stages. Results for CKD G5 should be interpreted with extreme caution due to small sample size (*n* = 61) and wide confidence intervals.

Our analysis revealed distinct patterns in the dose-response relationship between plant protein ratio and eGFR across CKD stages (Figure 4). In the Non-CKD group, the relationship demonstrated a U-shaped curve, with the lowest eGFR (90.66 mL/min/1.73 m^2^) observed at a plant protein ratio of 0.34, while the highest eGFR (91.84 mL/min/1.73 m^2^) was recorded at a plant protein ratio of 1.00. For CKD G2, similar to Non-CKD group, the lowest eGFR (72.15 mL/min/1.73 m^2^) was observed at a plant protein ratio of 0.31, and the highest eGFR (73.41 mL/min/1.73 m^2^) was found at a plant protein ratio of 1.00.

In CKD G3, the relationship exhibited an M-shaped curve. The lowest eGFR (49.47 mL/min/1.73 m^2^) occurred at a plant protein ratio of 0.78, while the highest eGFR (50.15 mL/min/1.73 m^2^) was observed at a plant protein ratio of 0.35. For CKD G4, we observed an inverted U-shaped curve. The eGFR reached its peak (25.60 mL/min/1.73 m^2^) at a plant protein ratio of 0.57, while the lowest eGFR (22.38 mL/min/1.73 m^2^) was found at a plant protein ratio of 1.00. In CKD G5, the dose-response relationship demonstrated a clear U-shaped curve, with the lowest eGFR (7.15 mL/min/1.73 m^2^) at a plant protein ratio of 0.25. The highest eGFR (12.98 mL/min/1.73 m^2^) was observed at a plant protein ratio of 0.72. These findings suggest that the optimal plant protein ratio varies across different CKD stages, with distinct patterns of association observed at each stage.

### Dose-response relationship between plant protein ratio and eGFR or all-cause mortality across CKD stages

**FIGURE 5:**
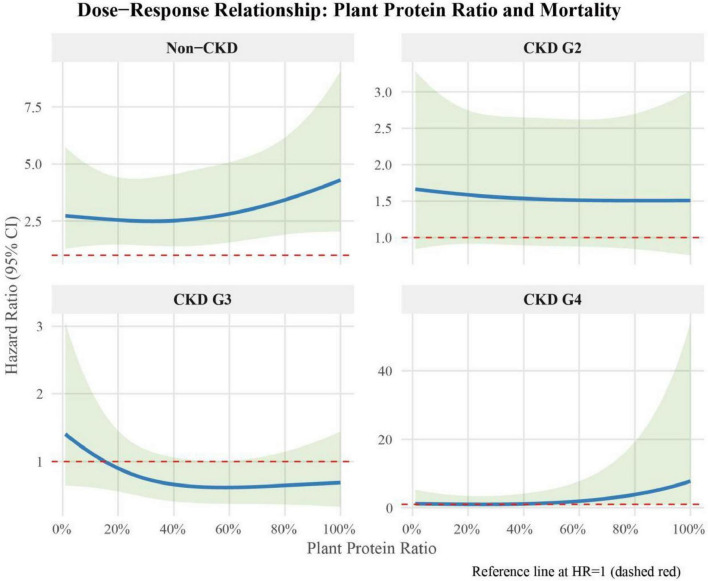
Dose-response relationship between plant protein ratio and all-cause mortality across CKD stages.

The dose-response relationship between plant protein ratio and all-cause mortality varied significantly across different CKD stages (Figure 5). In the Non-CKD group, we observed a U-shaped relationship. The lowest mortality risk (HR = 2.49, 95% CI: 1.41–4.41) was observed at a plant protein ratio of 0.33. The mortality risk increased gradually both below and above this point, reaching HR = 4.30 (95% CI: 2.04–9.07) at a plant protein ratio of 1.00. For CKD G2, the relationship demonstrated a relatively flat curve. The lowest mortality risk (HR = 1.51, 95% CI: 0.87–2.62) occurred at a plant protein ratio of 0.57, with minimal variations across different ratios. Even at the highest plant protein ratio of 1.00, the HR only slightly increased to 1.51 (95% CI: 0.76–3.02). In CKD G3, we observed an inverse U-shaped relationship. The mortality risk decreased as plant protein ratio increased up to 0.56, reaching its lowest point (HR = 0.62, 95% CI: 0.38–1.01), then showed a slight increase. At a plant protein ratio of 1.00, the HR was 0.69 (95% CI: 0.33–1.44). For CKD G4, the relationship showed a J-shaped curve. The lowest mortality risk (HR = 1.01, 95% CI: 0.30–3.37) was observed at a plant protein ratio of 0.25. Beyond this point, the mortality risk increased dramatically, reaching HR = 7.80 (95% CI: 1.13–54.09) at a plant protein ratio of 1.00. Due to the limited sample size in CKD G5 which prevented independent modeling, and considering the similar clinical characteristics and dietary management strategies in advanced CKD stages, we combined CKD G4 and G5 for a more robust analysis. For advanced CKD (G4-5), the relationship showed a J-shaped curve (Supplementary Figure 5). The lowest mortality risk (HR = 0.97, 95% CI: 0.38–2.47) was observed at a plant protein ratio of 0.31. Beyond this point, the mortality risk increased dramatically, reaching HR = 1.65 (95% CI: 0.2–13.56) at a plant protein ratio of 1.00. These findings suggest that the optimal plant protein ratio for mortality risk varies across CKD stages, with moderate plant protein intake generally associated with better survival outcomes in most stages. The relationship becomes more pronounced in advanced CKD stages, particularly in advanced CKD.

## Discussion

Dietary protein management in CKD requires careful consideration of both quantity and source. Plant-based proteins may offer advantages over animal proteins through multiple mechanisms, including lower acid load, different amino acid profiles, reduced uremic toxin generation, and lower saturated fat content. Additionally, plant-based diets typically provide higher fiber content and generate fewer advanced glycation end-products (14, 15). There are no one-size fits all nutrition prescriptions. Rather than adopting a universal approach, dietary recommendations should be tailored to individual patients based on their CKD stage, metabolic status, and specific cardiovascular risk factors (16). This personalized approach becomes particularly crucial as kidney function declines, where the balance between adequate nutrition and disease management becomes increasingly complex (17).

Our study provides novel insights into the stage-specific associations between plant protein ratio and both renal function and mortality in CKD patients. The key findings demonstrate that: (1) the relationship between plant protein ratio and eGFR exhibits distinct patterns across CKD stages, though statistical significance was limited to specific stage-ratio combinations; (2) optimal plant protein ratio ranges may vary by CKD stage, but confidence intervals were often wide, particularly in advanced stages; and (3) most associations between plant protein ratio and mortality risk did not reach statistical significance across CKD stages, with only CKD G3 medium plant protein ratio showing a borderline significant protective effect (*p* = 0.047) and CKD G4 high plant protein ratio showing increased risk (*p* = 0.025).

In early CKD stages (Non-CKD and G2), we observed a U-shaped relationship between plant protein ratio and eGFR, with lowest eGFR around 33%. This finding aligns with the physiological benefits of plant protein in early kidney disease, likely mediated through reduced acid load and inflammatory modulation (18–20). The improved eGFR associated with moderate plant protein intake may reflect the combined benefits of lower phosphorus bioavailability and reduced uremic toxin production compared to animal protein sources, while still maintaining adequate essential amino acid intake (21). However, the diminishing benefits at very high plant protein ratio (> 66%) suggest potential limitations of exclusive plant protein reliance, possibly due to reduced protein efficiency or micronutrient bioavailability.

For CKD G3, our analysis found no significant associations between plant protein ratio and eGFR. However, in terms of mortality risk, medium plant protein ratio (33%–66%) was associated with significantly lower risk compared to low plant protein ratio. A similar protective trend was observed in the high plant protein ratio group, though this did not reach statistical significance. These findings suggest that moderate plant protein intake might be beneficial for survival in CKD G3 patients, though the borderline significance and relatively wide CIs indicate the need for larger studies to confirm these observations. This stage-specific pattern adds to our understanding of how protein source effects may vary across different phases of kidney disease.

In CKD G4, we observed that medium plant protein ratio was significantly associated with higher eGFR compared to low plant protein ratio. However, the relationship with mortality was more complex. While our analysis suggested increased mortality risk with high plant protein ratio, the wide CIs and limited sample size in advanced CKD stages warrant cautious interpretation of these findings. Several potential mechanisms might explain these observations, including: (1) protein-energy wasting concerns in advanced CKD; (2) possible reduced protein utilization efficiency in severe kidney dysfunction; and (3) the inherently different nutritional profiles of plant versus animal proteins (22–25).

For CKD G5, although we observed numerical differences in eGFR across plant protein ratio groups, the small sample size limited our ability to draw firm conclusions. The mortality analyses in this group were similarly limited by sample size and statistical power.

These findings have potential implications for dietary management across the CKD spectrum, though additional research is needed to establish definitive recommendations. For early-stage patients (G1-3), moderate plant protein intake might be considered as part of overall dietary management (26). However, in advanced CKD (G4-5), our results suggest the need for careful individualization of protein sources, with close monitoring of nutritional status. We recommend regular monitoring of key nutritional biomarkers in advanced CKD patients consuming plant-based diets, including: serum albumin and prealbumin, nitrogen balance, inflammatory markers such as C-reactive protein, anthropometric measures, functional assessments, and uremic toxin levels. Additionally, phosphorus and potassium monitoring is essential given the typically higher mineral content of plant-based proteins.

The observed relationships in our study may help inform future research directions, though they should not be viewed as definitive guidelines. We suggest regular monitoring of nutritional status, particularly in advanced CKD patients consuming high-plant protein diets. Key indicators might include serum albumin, prealbumin, and nitrogen balance, alongside traditional markers of kidney function (27).

The paradoxical positive association between age and eGFR in CKD G4 likely reflects survival bias inherent to cross-sectional study design in advanced CKD populations. Older patients who reach CKD G4 represent a highly selected survivor population with potentially protective characteristics, while younger CKD G4 patients may represent more aggressive disease phenotypes with faster progression rates, resulting in lower eGFR values within the G4 range. Additionally, older patients may be diagnosed earlier in the G4 stage due to more frequent medical monitoring, whereas younger patients might be identified later when eGFR has declined further. This counterintuitive relationship underscores the importance of interpreting our CKD G4 results with particular caution and emphasizes the critical need for prospective longitudinal studies to establish true causal relationships between age, dietary interventions, and kidney function in advanced CKD.

The racial disparities observed in our study warrant careful consideration, as they reflect inequities in CKD burden and outcomes. Our findings showed that Non-Hispanic Black and Asian participants had consistently lower eGFR values across CKD stages compared to Non-Hispanic White participants, even after adjusting for traditional risk factors. This aligns with extensive literature demonstrating that Black Americans have a 2.5-fold higher cumulative incidence of kidney failure compared to White Americans, largely driven by younger age at CKD onset and higher progression rates (28). Among people with diabetes, Native Hawaiian/Pacific Islander populations have the highest CKD incidence rates (88.0 per 1,000 person-years), followed by American Indian/Alaska Native (85.2), Black (74.3), Hispanic (66.4), Asian (63.1), and White (47.3) populations, highlighting the disproportionate burden across racial and ethnic minorities (29). The differential effects of plant protein across racial groups may reflect several interconnected factors. First, genetic variations in protein metabolism and kidney disease susceptibility may modify dietary responses, as evidenced by differences in APOL1 gene variants that affect kidney disease risk predominantly in individuals of African ancestry (30, 31). Second, socioeconomic disparities significantly impact both food access and quality of plant protein sources, with minority populations having reduced access to high-quality plant proteins and greater reliance on processed foods (32, 33). Third, cultural dietary patterns differ substantially across racial groups, with recent NHANES data demonstrating that Mexican-American and other Hispanic females consume significantly higher levels of legumes, while Non-Hispanic Asian females show higher consumption of prudent foods including fruits, vegetables, and whole grains, and Non-Hispanic White and Black females exhibit higher consumption of cured meats, suggesting divergent baseline protein intake patterns that may influence dietary intervention responses (34). Fourth, the higher prevalence of diabetes and hypertension in Black and Hispanic populations may modify the relationship between dietary protein and kidney outcomes (35, 36). Our finding that protective mortality effects were more apparent in White and Other Hispanic participants may reflect these complex interactions between race, diet quality, healthcare access, and underlying disease burden. Importantly, research has demonstrated that when people from different racial groups have equal access to high-quality diabetes and CKD care, disparities in kidney outcomes begin to disappear, as evidenced by the 54% reduction in end-stage kidney disease rates among Alaska Native and American Indian populations treated through comprehensive community-based interventions (29). These disparities underscore the critical need for culturally tailored nutritional interventions and highlight how structural inequities in healthcare and food systems may influence the effectiveness of dietary recommendations across different populations.

Several important limitations of our study must be acknowledged. First, its cross-sectional design precludes causal inference regarding plant protein ratio and outcomes. Second, dietary data from 24-h recalls may not fully capture long-term intake patterns or seasonal variations. Third, the relatively small sample size in advanced CKD stages, particularly G5, substantially limited our ability to draw conclusions about these populations. Fourth, the choice of cut-points was data-driven based on tertile distribution in our population rather than established clinical guidelines. Future research should aim to establish evidence-based thresholds for plant protein ratios that could inform clinical practice guidelines for CKD management. Fifth, the interpretation of our findings is further limited by the high degree of statistical uncertainty in advanced CKD stages, as evidenced by wide CIs that often spanned both protective and harmful effects. This uncertainty, combined with small sample sizes, prevents definitive conclusions about optimal plant protein ratios in CKD G4 and G5 patients. Additionally, while we adjusted for multiple confounders, residual confounding from unmeasured variables cannot be excluded.

Future research should prioritize prospective studies examining the long-term impact of different plant protein ratios across CKD stages. Particular attention should be paid to protein quality metrics, amino acid profiles, and their interaction with disease progression. Randomized controlled trials comparing isocaloric diets with varying plant protein ratios in advanced CKD would help establish causality and optimal ranges. Additionally, investigation of potential effect modifiers such as age, comorbidities, and baseline nutritional status could further refine dietary recommendations.

In conclusion, our findings suggest that the relationship between plant protein intake and outcomes in CKD may vary by disease stage, though many of these associations require confirmation in larger, prospective studies. The results highlight the complexity of protein source effects in CKD and support the need for individualized approaches to dietary protein management. Future research should focus on validating these findings in prospective studies and clarifying the optimal role of plant protein across different CKD stages.

## Data Availability

Publicly available datasets were analyzed in this study. This data can be found here: https://www.cdc.gov/nchs/nhanes/.
