# Supplementary material for: Differential effects of plant protein ratio on renal function and mortality across CKD stages
Source: Front Nutr. 2025 Jul 28;12:1596836. doi: 10.3389/fnut.2025.1596836 (PMC12336025; doi:10.3389/fnut.2025.1596836)
Supplement: Supplementary file 1 [file Image_1.pdf]

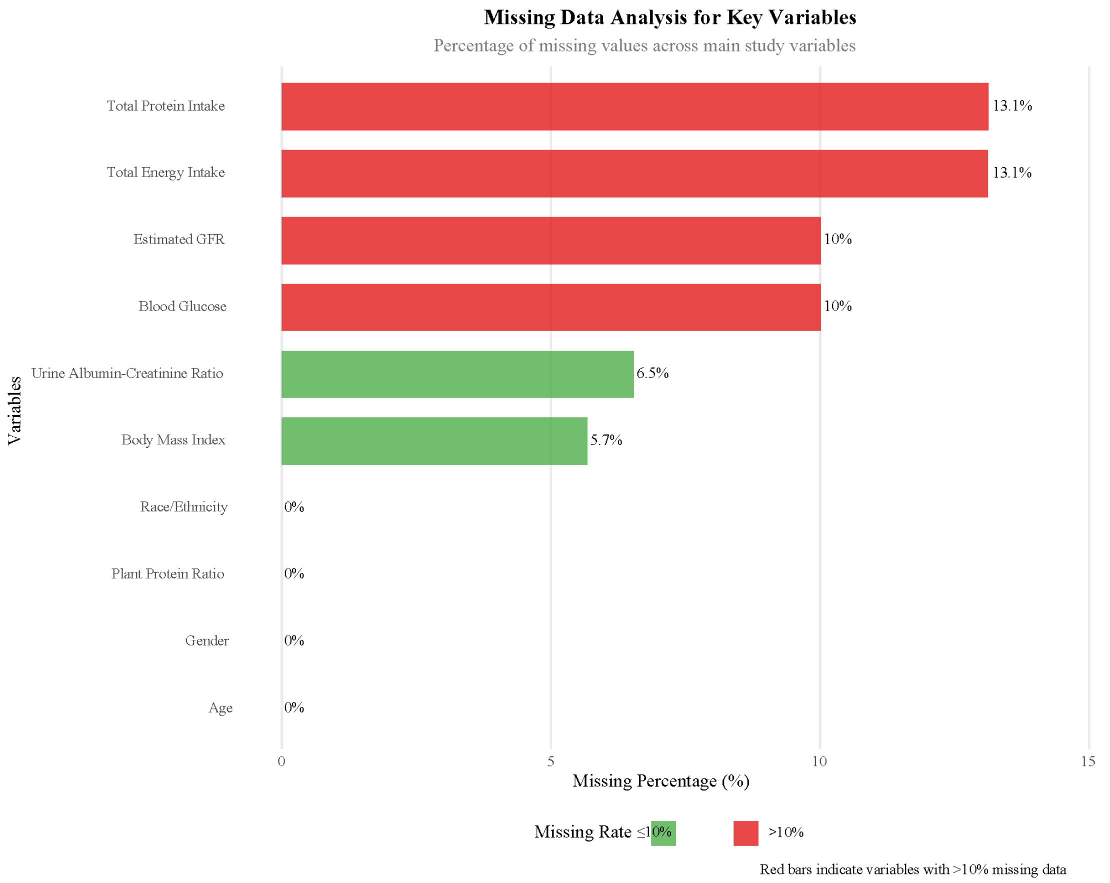

Supplementary Figure 1. Percentage of missing data for key variables.

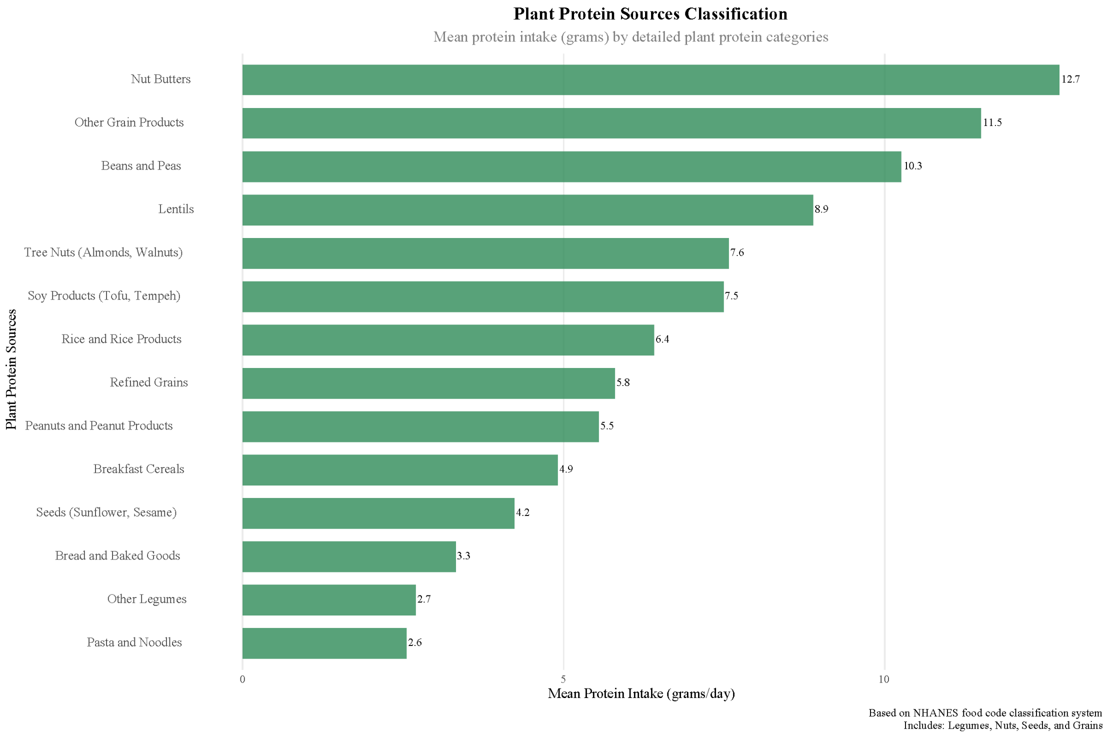

Supplementary Figure 2. Comprehensive list of plant protein source classifications.

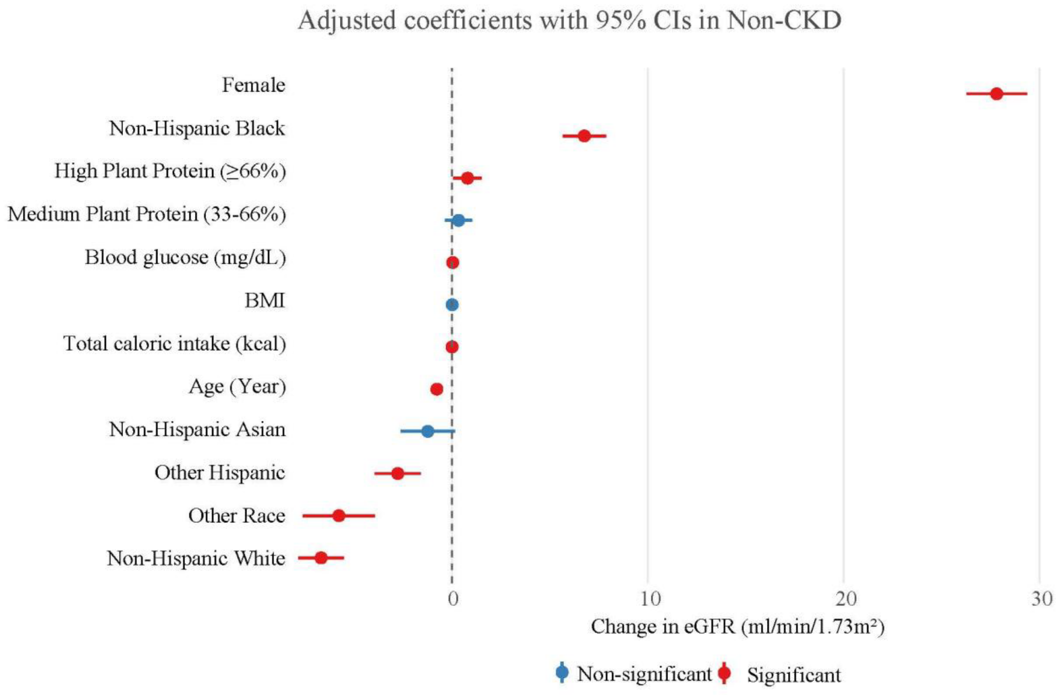

Supplementary Figure 3. Association between plant protein ratio and eGFR in Non-CKD.

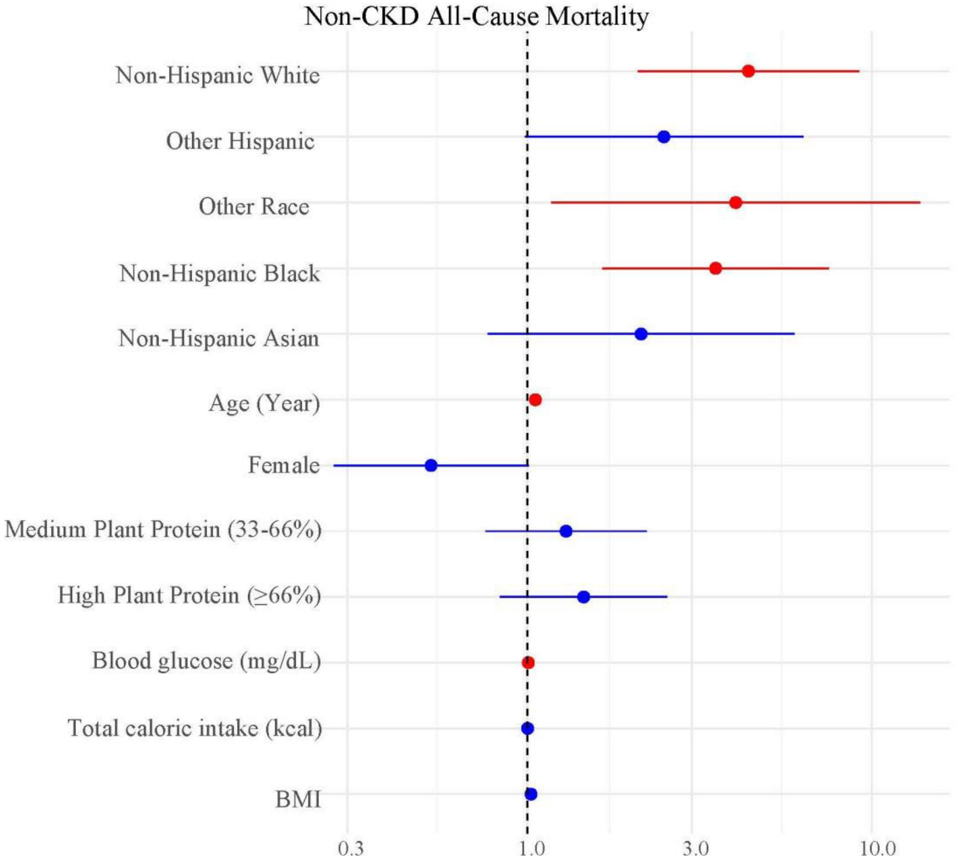

Supplementary Figure 4. Association between plant protein ratio and mortality risk in Non-CKD.

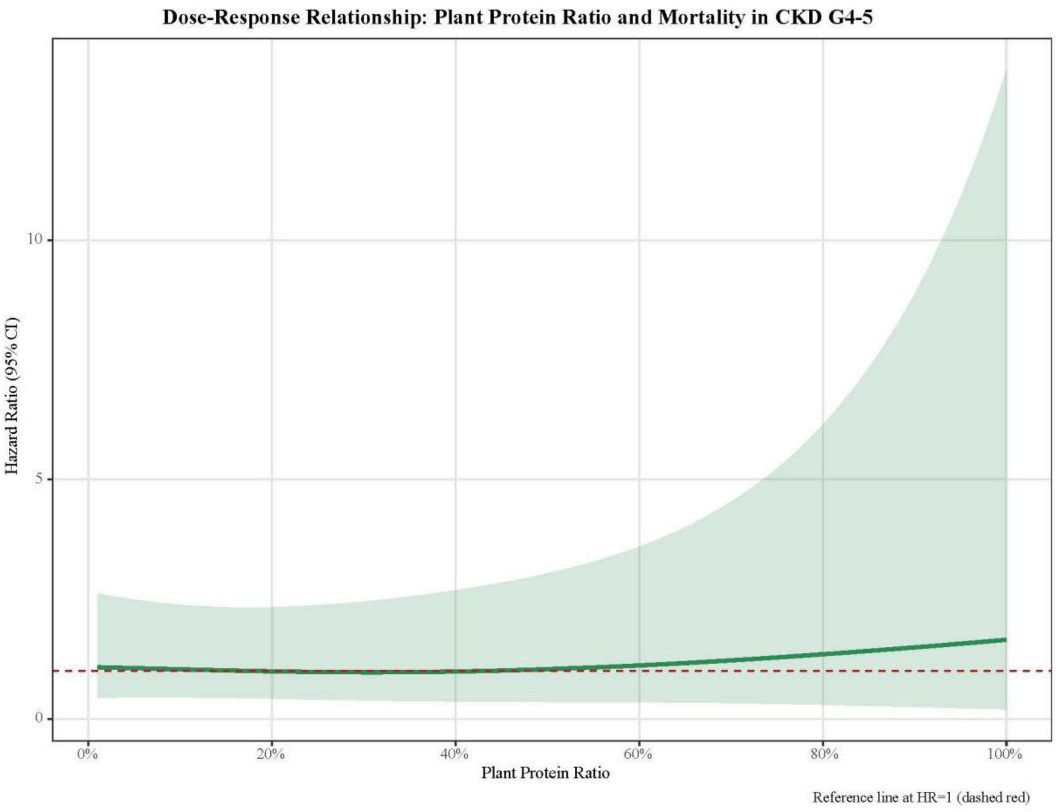

Supplementary Figure 5. Dose-response relationship between plant protein ratio and all-cause mortality in non-CKD.

Note: Results for CKD G5 should be interpreted with extreme caution due to small sample size (n=61) and wide confidence intervals.
